# Occupational therapists’ application of the Do-Live-Well framework: A Canadian health promotion approach

**DOI:** 10.1177/00084174221117717

**Published:** 2022-08-22

**Authors:** Sungha Kim, Nadine Larivière, Ilana Bayer, Rebecca Gewurtz, Lori Letts

**Keywords:** Health promotion, Knowledge application, Occupational-focused approach, Application des connaissances, approche axée sur les occupations, promotion de la santé

## Abstract

**Background.** The Do-Live-Well (DLW) framework is an occupation-focused health promotion approach. Online and in-person DLW educational workshops were offered to encourage occupational therapists to apply the DLW concepts. **Purpose.** The purpose of this study was to understand workshop participants’ experiences of and perspectives on using the DLW framework to support its application in the future. **Method.** Interpretative description was used to understand workshop participants’ perspectives on benefits, facilitators, and challenges of using DLW. Semi-structured interviews were conducted and analysed using a thematic analysis. **Findings.** Eight themes were identified as follows: (a) environmental factors of practice settings, (b) co-workers’ support, (c) DLW enhanced occupational therapy practice, (d) confidence in using DLW, (e) nature of the DLW framework, (f) DLW promoted healthy occupational engagement, (g) DLW was not suitable for everyone, and (h) pandemic effects. **Implications.** The DLW framework supports occupationally focused practices, and continuous learning support will be needed.

## Résumé

**Description.** Le modèle « Vivez bien votre vie » (VBVV) est une approche de promotion de la santé axée sur les occupations. Des ateliers de formation en ligne et en personne ont été offerts pour encourager les ergothérapeutes à appliquer les concepts de VBVV. **But.** Le but de cette étude est de comprendre les expériences et les points de vue des participants à l’atelier quant au modèle VBVV en vue de ses futures applications. **Méthodologie.** Les points de vue des participants à l’atelier quant aux avantages, aux éléments facilitateurs et aux défis de l’utilisation du modèle VBVV ont fait l’objet d’une description interprétative. Des entrevues semi-structurées ont été réalisées et ont fait l’objet d’une analyse thématique. **Résultats.** Les huit thèmes suivants ont été recensés : (a) les facteurs environnementaux des différents contextes de pratique, (b) le soutien des collègues, (c) l’enrichissement de la pratique de l’ergothérapie par VBVV, (d) la confiance dans l’utilisation de VBVV, (e) la nature du modèle VBVV, (f) la promotion de l’engagement occupationnel sain par l’entremise de VBVV, (g) le fait que VBVV ne convient pas à tous et (h) les effets de la pandémie. **Conséquences.** Le modèle VBVV soutient les pratiques axées sur les occupations, et un soutien continu dans son application sera nécessaire.

## Introduction

Health promotion is a growing part of the occupational therapy scope (Söderback, 2015), and occupational therapists’ roles in health promotion include “enabling, mediating and advocating to build healthy public policy; creating supportive environments; strengthening community action; developing personal skills; and reorienting health services” (Letts et al., 1993, p. 10). Although it has been established that occupations can promote individuals’ health and wellness (Creek & Hughes, 2008; Law et al., 1998; Wilcock & Hocking, 2015), health promotion in Canada has focused primarily on diet, exercise, medical checkups, and smoking (Nettleton, 2021). In addition, there is a lack of guiding models or theories to support occupational therapists’ incorporation of health promotion concepts in their practice (Hildenbrand & Lamb, 2013). Thus, an occupation-focused health promotion framework was needed to facilitate health and well-being for Canadians of all ages and abilities.

The Do-Live-Well (DLW) framework is a health promotion approach developed by the Canadian occupational therapists. The main idea of the framework is that “what you do every day matters,” and the framework was developed to convey the message to individuals that they can have “choices and opportunities for living well” (Moll et al., 2015, p. 11). The DLW framework was designed for use by any individual, group, or community regardless of age and health status (Moll et al., 2015). The DLW framework comprises four main constructs: dimensions of experience, activity patterns, social and personal forces, and health and wellness outcomes. The framework reflects participating in diverse experiences with optimal activity patterns and sufficient personal and social support, which can result in a wide range of positive health and wellness outcomes (Moll et al., 2015). The components of each section of the framework are described in Table 1. The section of dimensions of experience describes eight broad dimensions of everyday experiences that affect health and wellness (Moll et al., 2015). For example, participating in volunteering activities that contribute to the community and society are related to lower rates of depression and improved perceived health (Gottlieb & Gillespie, 2008; Grimm et al., 2007). The activity patterns section explains that how people engage in their daily activities also affects health and wellness (Moll et al., 2015). Next, the DLW framework outlines individual and social factors that affect people's occupational engagement (Moll et al., 2015). Finally, the framework provides a broad view of health and wellness by focusing on emotional, social, and spiritual health in addition to physical and mental health (Gewurtz et al., 2016; Moll et al., 2015). The DLW framework emphasizes the importance of participating in diverse meaningful experiences for better health and well-being. Although this framework is neither a model of practice nor prescriptive, it may provide occupational therapists with a structure to consider how they can contribute to healthy communities and develop tools or interventions that incorporate occupations in practice. However, this relatively new framework has not been widely adopted by occupational therapists, perhaps because of a lack of opportunities to disseminate the knowledge surrounding this framework. The DLW team has therefore offered educational opportunities for occupational therapists in recent years. As part of this knowledge dissemination effort, a research project compared the effectiveness of in-person and online workshops (Kim et al., 2022). Although the project allowed the researchers to understand the effectiveness of both educational methods and the participants’ perspectives regarding their workshop experience, it did not identify how workshop participants used the framework and what challenges, facilitators, and benefits of applying the DLW framework they experienced in practice. It is important to understand which aspects of the framework worked and which did not in different practice settings to support the adoption of this framework for occupational therapists in the future. A study by Kim et al. (2022) reported no difference in knowledge gained regarding the DLW framework between the online and in-person workshop groups; thus, this project's focus was to explore how the workshop participants applied the DLW framework, regardless of the format of workshop delivery. This study aimed to understand the participants’ perspectives on using or not using the DLW framework and what worked and what did not work for their practices. The research question was “What are the occupational therapists’ perspectives on the application of the DLW framework in practice after participating in a workshop?”

**Table 1:** Components of Each Section of the Do-Live-Well (DLW) Framework.

Section	Components
Dimensions of Experience	Activating your body, mind, and senses
	Connecting with others
	Contributing to community and society
	Taking care of yourself
	Building security/prosperity
	Developing and expressing identify
	Developing capabilities and potential
	Experiencing pleasure and joy
Activity Patterns	Engagement
	Meaning
	Balance
	Control/Choice
	Routine
Social and Personal Support	Personal forces: age, gender, ethnicity, health, etc. Social forces: accessibility, affordability, restrictive rules, etc.
Health and Wellness Outcome	Physical, mental, social emotional, spiritual health, and wellness

## Method

### Design

This study used Thorne’s (2016) interpretative description methodology to understand participants’ perceived challenges and benefits of applying the DLW framework after they participated in a workshop about the DLW framework. An interpretative description provides researchers with a flexible method to interpret participants’ experiences and apply the findings to practice (Thorne, 2016). This methodology was considered appropriate for this study because the researchers wanted to understand how the DLW concepts could be used in occupational therapy practice and what to consider to support occupational therapists’ use of the framework. This study was approved by Hamilton Research Ethics Board.

### Participants

Participants were Canadian occupational therapists who attended online or in-person DLW workshop. We recruited participants who were from different practice settings and from both formats of the workshops. Regardless of whether they were currently using the DLW framework, participants were recruited to understand the comprehensive experience of the application of the DLW framework. The first author contacted all workshop participants (n = 50) to request their participation in a one-on-one interview 3 months after their completion of the workshop. The target sample size was 10–15 occupational therapists with the possibility to modify the sample size based on saturation in answering the research question (Guest et al., 2006). A written consent form was reviewed and signed by the participants once they agreed to the interview.

### Data Collection

The first author conducted individual, semi-structured interviews with each participant via the videoconference platform Zoom, and the interview was designed to take 40–60 min. The interview guide was developed prior to the interviews by the first author and verified by the research team to explore the individual's experience with the application of the DLW framework in practice during and after workshop participation. The primary focus was on the benefits, facilitators, and challenges of its use in practice. It included 15 open-ended questions on the following three topic areas: (a) how respondents used the DLW framework with their clients; (b) benefits/challenges of using the DLW framework with clients; and (c) benefits of using the DLW framework as an occupational therapist.

### Data Analysis

The data were analysed using a thematic analysis approach (Braun & Clarke, 2006). All interview records were transcribed, and the first author read the transcriptions several times and identified codes in both large and small chunks of the data, which were relevant to the idea of benefits, facilitators, and challenges of using the DLW framework in practice. Emerging codes from new transcripts were compared to the existing codes, and similar codes were organized together to create themes. The initially identified themes were reviewed by all authors to increase the trustworthiness of the findings, and the authors discussed whether the identified codes and themes were appropriate and answered the research question. The themes were then refined and finalized by the first author. Since the purpose of this study was to obtain a broad understanding of the use of the DLW framework in practice, we did not compare participants’ perspectives and experiences based on whether they used the DLW framework or not. Some of the researchers were the core members of the DLW team, which allowed us to gain a deep understanding of which aspects of the DLW framework worked well and which did not work well in occupational therapy practice.

#### Rigour of the findings

While member checking is used to ensure the credibility of qualitative findings (Merriam, 2009), it was not recommended in the interpretative description methodology as the emphasis of this methodology is on the importance of researchers’ interpretation (Thorne, 2016). Rather than performing member checking, the first author summarized the ideas and wrote a reflection note related to each participant interview, and critical decisions made during the analysis were recorded in the reflective journal to enhance the credibility of the findings (Thorne, 2016). The first author also had several discussions with the research team regarding the process of conducting the analyses and the emerging findings (Merriam, 2009).

#### Use of a multilevel framework influencing the adoption of new knowledge

While analysing the interview transcripts, we recognized levels of factors that influence the adoption of the DLW framework in practice. Therefore, as part of the interpretive stage of analysis, we decided to incorporate a multilevel framework that accounts for the different levels of factors that influence implementation outcomes (Chaudoir et al., 2013). These levels include structural, organizational, provider (occupational therapists), innovation (the DLW framework), and patient (client) factors. The structural level consists of factors related to the physical environment, policies, and social and economic situations of the implementation site; the organizational-level factors include the degree to which an organization values new knowledge; the provider-level factors include the users’ attitudes towards the knowledge implementation; the innovation-level factors include the benefits of implementing new knowledge; and the patient-level factors are patients’ characteristics that can affect the implementation of knowledge. In addition to the levels described by Chaudoir et al. (2013), we added an unexpected factor caused by the coronavirus disease 2019 (COVID-19) pandemic. The multilevel framework (Chaudoir et al., 2013) was not used as a priori for the inductive analysis but emerged through second-level analysis as a useful theming framework to provide a structure for presenting our findings.

## Findings

### Participants’ Characteristics

Eighteen occupational therapists, (in-person: n = 9, online: n = 9), comprising 17 females and 1 male, from different practice settings participated in interviews. The mean age of the participants was 39.56 (SD = 9.95), and the mean of their years working in occupational therapy was 13.44 (SD = 9.57). Fourteen participants had their master’s degree in occupational therapy, and four had a bachelor's degree in the field.

The participants’ self-described practice varied, as follows: mental health (n = 6), primary care (n = 2), hospital (n = 2), education (n = 1), long-term care (LTC) (n = 1), ophthalmology clinic (n = 1), paediatrics (n = 1), accessibility (n = 1), private practice (n = 1), rehab unit (n = 1), and veterans’ centre (n = 1).

Ten occupational therapists reported they were using DLW concepts in their practice, whereas eight said that they had not applied the DLW framework since their participation in the training workshop.

### Themes

Participants shared their experiences and perspectives on applying the DLW framework in their practices. Eight themes were identified according to six levels of factors that influenced the adoption of the DLW framework (Table 2).

**Table 2:** Identified Themes Under six Levels of Factors Influencing new Knowledge Adoption.

Factor Level	Theme
Structural	• Environmental Factors of Practice Settings Affected • The Application of the DLW Framework
Organizational	• Co-workers’ Support of Using DLW
Provider (Occupational Therapists)	• The DLW Framework Enhanced Occupational Therapy Practice • Confidence in the use of the DLW Framework Affected its Application
Innovation (The DLW Framework)	• Nature of the DLW Framework Prevented or Facilitated its Application
Patient (Client)	• DLW Concepts Promoted Clients’ Healthy Occupational Engagement • The DLW Framework was not Suitable for Everyone
Unexpected	• Pandemic Effects on the Application of the DLW Framework

DLW = Do-Live-Well.

### Structural-Level Factors

#### Environmental factors of practice settings affected the application of the DLW framework

Occupational therapists in various practice settings participated in this study, and the availability of resources (e.g., human, time, and physical space) differed depending on their practice settings. The practice system and the availability of resources in each setting affected many participants’ applications of the DLW framework.

Occupational therapists shared how limited time spent with their clients affected their use of the DLW framework. For example, one participant also explained that, in an acute setting where there is a rapid cycle of clients coming and going, there was little time to apply the concepts and principles from the framework. However, this feeling of a lack of time may be because this participant thought that the application had to be complete, using the entire DLW framework. This participant said, “There were questionnaires, evaluation tools…I found that there really was not a lot of time to fit them into the process” (Interview 10).

Another participant in an education department who previously worked in a mental health setting said that it would be hard to apply the DLW framework in outpatient settings owing to a lack of time spent with clients. She said, “One of the neat things about [the DLW framework] is you can apply aspects of it probably everywhere to a certain degree, but you could probably apply it more fulsomely when you have more time with a client” (Interviewee 15).

Thus, regardless of the types of practice settings, the idea that many or all aspects of the DLW framework should be used in practice may have led participants to think that there is not enough time to use this framework.

In addition, the available physical space where clients could participate in various activities in a practice setting affected occupational therapists’ DLW application in practice. Some practice settings were well equipped to allow clients to engage in a wide range of activities, such as gardening and group exercise, during their stay at the institution or facility, enabling occupational therapists to incorporate the DLW concepts by supporting clients’ engagement in different dimensions of experiences. A participant who used the DLW framework in a veterans’ centre said,

I think it is the facility that I work in [that enables the application of DLW]. I mean … they live here, and there are so many resources available to them. I think that really helps, and not looking outside of this building necessarily for activities as we just have such a huge recreational therapy department, creative arts therapy. (Interviewee 5)

Insufficient physical space in practice settings negatively affected the application of the DLW framework. One participant (Interviewee 18) working in a mental health setting who did not use the DLW framework reported her clinical practice setting lacked space for group programs, which was how she wanted to use the DLW framework with her clients.

The availability of human resources also affected the application of the DLW framework. The presence or absence of someone who could help with the application of DLW concepts in treatment affected the use of the DLW framework. For example, if a client who is not mobile without assistance wants to go outside to engage in some enjoyable activities, such as gardening or going to see a movie, there needs to be a person who can help the client get outside and assist them in those activities (Interviewee 11).

One participant said that, thanks to the presence of student occupational therapists, she was able to collaborate with them to apply the DLW framework:

I was having a student at the same time [who] did the workshop. I talked with the student about [DLW] and worked with [the student] to see how we could incorporate it into an initial assessment that [clients] had, and how [we] could frame the treatment around using the Do-Live-Well framework. It was the both of us working together on how to use it. (Interviewee 3)

Having a collaborator and someone with available time was helpful for this occupational therapist to incorporate the framework into practice.

### Organization-Level Factors

#### Co-workers’ support of using DLW

As an organization-level factor, it was important for participants to have the support of their co-workers in using the DLW framework and putting new knowledge into practice. Some participants said their team members were supportive of their use of the framework because they respected their colleagues’ work and understood the importance of occupational therapy:

I can use it because they trust me to do whatever I do in occupational therapy as long as my goals that I am talking about line up with the team…or I can explain why this is important to the client. (Interviewee 16)

On the other hand, if co-workers are not supportive or do not appreciate the DLW concepts, it might be difficult for occupational therapists to use the DLW framework. One participant in an ophthalmology clinic said, “If the rest of my team is not looking at [clients] through that lens, it could be a little bit more challenging to implement” (Interviewee 14). Thus, co-workers’ attitude towards new knowledge can affect the adoption of new frameworks in practice. However, co-worker support was not the only factor that influenced the application of the DLW framework. Even with the support of one's colleagues, there still may be other barriers to implementation. One participant said, “I work in a really supportive workplace, so I think they would absolutely support the implementation” (Interviewee 11). However, she expressed she was not yet ready to use it owing to her lack of confidence in using the DLW framework correctly. This demonstrates why it is important to understand different factors that influence framework application. Although one factor may encourage a participant to apply the DLW framework, other factors can affect their adoption of new knowledge and their decision on whether to ultimately implement it.

### Provider-Level Factors

#### The DLW framework enhanced occupational therapy practice

Many participants valued the DLW framework concepts and believed using them would improve their practice regardless of whether they used the DLW framework or not. First, they valued how the DLW framework emphasizes the importance of occupation in people's health and well-being, which is the core value of occupational therapy. One participant who did not use the DLW framework said, “I think it [DLW] just really grounds us in the benefits of occupation [and] just gets back to what occupational therapy is about, which is the benefits of meaningful occupation” (Interviewee 1).

Furthermore, participants identified that the DLW provides a new way to talk and think about occupation, occupational therapy, and what occupational therapists can do to support their clients’ health and wellness. By introducing and explaining the different types of occupations described in DLW to their clients, occupational therapists helped their clients better understand what occupations are.

Some participants especially valued the client-centredness of the DLW framework. By empowering the client to identify and choose the occupation that could better promote their health, the DLW framework appeared to increase clients’ voices in terms of priority setting. One participant who did not use the DLW framework said, “It is really about being client-centred like we originally professed to be, and finding out what is important from a client's point of view…looking at what [our clients are looking for] in terms of [their] health and wellness” (Interviewee 10).

Finally, the DLW framework was a tool that can be incorporated as part of a holistic approach to occupational therapy. One participant said,

I think it really enables me to actually practice much more holistically…The Do-Live-Well framework is a very concrete way of at least acknowledging the whole person and that, aside from their basic ADLs [Activities of daily living], there are many more dimensions of experiences that are equally meaningful. (Interviewee 11)

#### Confidence in the use of the DLW framework affected its application

Along with the belief that the DLW framework could improve their practice, participants’ confidence in their knowledge of the DLW framework also influenced their application. One participant using the DLW framework reported that she used it mainly because she knew the DLW concepts well enough to translate them into the practice. She said, “I am more comfortable with the concepts and how to incorporate them into the things that we talk about with my patients” (Interviewee 3).

A participant who did not use the DLW framework said the primary reason she did not use the DLW concepts was because she was unsure if she could correctly use it (Interviewee 11). Thus, she lacked confidence in her knowledge of the DLW concepts.

### Innovation-Level Factors

#### Nature of the DLW framework prevented or facilitated its application

Some of the unique characteristics of the DLW framework seemed to affect occupational therapists' application of the DLW framework. These characteristics include various components, being non-prescriptive, being designed for any individual, and using occupational therapy language. Participants shared their perspectives on the nature of the DLW framework that affects their application of it in practice. First, the DLW framework consists of different components, and some occupational therapists felt it contains too much information and could be overwhelming. Thus, for occupational therapists who did not completely understand the DLW framework (even after participating in the workshop), it would be difficult for them to apply it: “Because I do not remember all [components of the DLW framework]… So that it is the only reason I do not think I am using the framework” (Interviewee 17).

Moreover, the DLW framework is not prescriptive-affected occupational therapists’ DLW application. Some occupational therapists found using DLW difficult as it is conceptual rather than procedural; there are no guidelines to explain steps, making it challenging to apply in practice. One participant said, “I was struggling in the workshop in terms of this is not a prescribed program, there's not a set step-by-step way to use it.” (Interviewee 4)

However, some participants valued that the DLW was designed to help anyone with any ability or any health condition at any age: “The other nice thing too is that the Do-Live-Well does not focus on ability or disability at all. It is applicable across the board” (Interviewee 11).

This scope allowed occupational therapists to adapt the concepts more efficiently in their practice: “It makes sense to apply these concepts for everyone. I think everyone can benefit from it” (Interviewee 6).

Interestingly, many participants applied the DLW concepts for themselves, family, and friends to promote their own health and wellness and their loved ones’, which once again emphasized that the framework is designed for anyone. One participant said, “I was thinking about it [DLW] even just in terms of my own daily life and I think it is a helpful framework for thinking about how much do you think about your own self” (Interviewee 15).

Additionally, one participant said applying the DLW framework to her own life could help persuade her clients to incorporate the DLW concepts in their daily life.

As an occupational therapist, I need to have to buy in first, right? I need to be able to have the lived experience first for me to be able to tell convincingly to my client [to use DLW]. (Interviewee 8)

Finally, the language used in DLW either facilitated or hindered its application. Canadian occupational therapists developed DLW, so words used in the DLW framework are very Occupational Therapy-driven; thus, it might be hard for some clients and co-workers unfamiliar with the occupational therapy language to understand the DLW concepts. Also, occupational therapists believed they would need to adjust the language based on the clients’ level of understanding: “I think people understand those types of terms, recognizing you are going to adjust it for anybody that you are talking to” (Interviewee 14).

### Client-Level Factors

#### DLW concepts promoted clients’ healthy occupational engagement

Regardless of whether the participants used the DLW framework, they valued its core concepts of promoting the health and wellness of individuals. Some participants said that the DLW framework helped their clients explore different areas of experience, allowing them to reflect on activities they lack in their daily life and enabling them to participate in various occupations related to their comprehensive health and wellness. One participant said, “I just think [the DLW framework] gives them the most opportunities to be able to participate in the activities to improve their health and their well-being” (Interviewee 5).

In addition, the DLW framework helped ensure that clients are engaged in occupations with appropriate activity patterns. If a client had an issue in their specific occupation, the five activity patterns specified in the DLW framework allowed occupational therapists to investigate how their clients are engaged in the occupation. One participant shared how the DLW concepts might be helpful in understanding her client's issues with sleep:

I think I would look into his activity patterns of the day. Look at what he's busy with, what kind of activities kind of helps him manage his tremors, what kind of routine he has in the evening times that might lead to restlessness at night. (Interviewee 13)

Also, for clients who feel overwhelmed by their occupations, the DLW's activity pattern concepts allowed them to reflect on whether they are engaged in their occupation in a balanced way. One participant said, “It's about mak[ing] sure that you’re maximizing your time and your day and your routine so that you are healthy, happy, and you feel like you’re working towards something” (Interviewee 6).

An occupational therapist who did not use the DLW framework also valued the framework because it may facilitate clients’ motivation to be involved in different activities that can be related to positive health outcomes. She said, “[the] Do Live Well framework increases [a] sense of satisfaction, levels of motivation perhaps, and [clients’] ability to actually engage in those activities as a means to recover from depressive symptoms beyond just activating” (Interviewee 18).

#### The DLW framework was not suitable for everyone

The status of clients affected the DLW application. Participants perceived little room for incorporating the DLW framework for clients whose basic [ADL] needs, such as toileting and bathing, had yet to be met or who functioned at lower levels. Children or clients with cognitive impairment could also have difficulty understanding the DLW concepts: “For our clients, I think it is their ability to understand the information [that determines the use of DLW]” (Interviewee 5). “All the examples that [DLW seems] to be giving were much more for adults. Like you lost your job, or you lost this, or this is happening. So, there was not as much concrete form of evidence [for young children]” (Interviewee 7).

As the DLW framework is client-centred, it requires clients’ engagement in determining gaps in their daily activities and discussing what they want to do to improve their health and wellness. However, clients may not participate in various activities or change their existing activity patterns due to the realities of choice, opportunity, priorities, and prior experiences. Then, it may be difficult for them to incorporate the DLW concepts. One participant said,

I think it really depends on a lot of factors but one of them is their motivation level… if they are not willing to give up certain behavioral patterns, then you cannot really force them, right? (Interviewee 8)

Some occupational therapists in this study have also had conflicting ideas about whether it is appropriate to use the DLW framework for certain populations. One participant in the LTC setting said it might not be appropriate to use the DLW framework in practice for people in forensic settings because they do not have much choice and control in participating in activities. However, an occupational therapist working in a forensic psychiatric setting said DLW concepts fit very well with her practice because her clients are often referred to her based on a lack of structure in their lives. Thus, the activity patterns gave her clients routines that promoted their health and wellness. She said, “To help them get structure in their life—help them start doing something. I think that the activity patterns fit very well with that, and then the rest of it though just kind of flows with it” (Interviewee 16).

### Unexpected Factor

#### Pandemic effects on the application of the DLW framework

Since COVID-19 began to spread in Canada in late 2019, many regulations and measures have been enacted to prevent the virus from spreading further. These restrictions changed practice contexts by reducing in-person interactions. This change could have negatively affected how many occupational therapists applied DLW. Given that the interviews were conducted in May 2020, during the first months of the pandemic, social distancing public health orders and movement restrictions related to reducing the spread of the virus, may have prevented occupational therapists from adding new knowledge to their standard routines, especially when their practice was not ready to transition their occupational therapy sessions from in-person to online. One participant expressed her frustration with the situation, saying,

I think right now with COVID it is difficult because [patients] are not interacting with people as much. A lot of the groups have been cancelled…I think it is hard right now to be able to implement more. (Interviewee 5)

In addition, it was not easy to apply some of the DLW concepts under the physical restrictions implemented during the pandemic. For example, “connecting with others” and “contributing to society” are most conducive to in-person environments, so it was difficult to participate in those activities with social distancing measures in place. One participant said,

But right now, it is going to be very difficult because of COVID. They will not be able to go out and do whatever they want to. If their goal, say, is to connect with their community, when, say, if they are living in a group home, they are in lockdown, they cannot go out, then there is no point. (Interviewee 8)

Additionally, owing to the severity of COVID-19, some occupational therapists were struggling with increased stress and responsibilities. Thus, the timing was not ideal to implement new ideas in practice. One participant said, “The fear of a novel virus and how am I going to keep myself and my family safe, it was such a huge cognitive load that I do not and literally could not process much of anything else” (Interviewee 11).

However, some occupational therapists found that COVID-19 facilitated their use of the DLW framework in their practice. They used the DLW framework to identify gaps in clients’ daily occupations in this isolated situation. One participant shared her ideas on how DLW was helpful in this pandemic: “Just particularly going through the dimensions of experience with the clients was really helpful at trying to find some gaps maybe in where they might be struggling in terms of their day-to-day health and occupations” (Interviewee 5).

Further, one occupational therapist developed a quick COVID-19 resource guideline based on the DLW framework:

I created a quick resource guide for COVID. So again, kind of using elements of the tools that are within the framework for people to kind of reassess or really reflect on how the current pandemic has changed their engagement in occupation. (Interviewee 2)

## Discussion

This qualitative study explored occupational therapists’ experience and perspectives on the application of the DLW framework in practice after participating in a DLW workshop. Consideration of each level of the factors influencing adoption allowed us to understand a wide range of factors that facilitated or hindered participants’ incorporation of the DLW framework. Qualitative data analyses resulted in eight themes concerning some benefits, facilitators, and challenges of the application of the DLW framework in occupational therapy practice. These results provide insight into what aspects of the DLW framework worked well or did not work well and what factors affected their use of the DLW framework, which may contribute to occupational therapy practice in applying the health promotion approach. Overall, the findings support the DLW framework's value in occupational therapy practice and provide the DLW research team with essential insights for promoting its use.

There were factors that facilitated participants’ implementation of the DLW framework in practice. First, the core concepts of the DLW framework allowed occupational therapists to reflect in-depth on occupations in their practice as a means of occupation-based practice. Occupational therapists’ ultimate goal is to promote people's overall health, wellness, and quality of life by encouraging them to participate in a variety of meaningful occupations (Hammell, 2017). This idea fits well with the core concept of the DLW framework: improving people's health and wellness by exploring the potential benefits of experiences derived from an occupational lens, with optimal activity patterns. Some participants of this study may have believed the DLW framework is helpful in their practice because of this close link to the core theory of occupational therapy; the DLW framework allows them to support their clients’ healthy occupational participation by identifying gaps in their daily life and exploring different dimensions of experience that could be related to positive health and wellness.

Next, study participants used the DLW framework because it can improve their occupational therapy practice as an added tool. Occupational therapists acknowledge different occupation-based theories, frameworks, or models so that they can choose one that is appropriate for their clients (Cole & Tufano, 2020; Duncan, 2020). Incorporating a framework into an occupational therapy practice can improve practice by supporting clinical reasoning (Boniface & Seymour, 2011). Thus, the DLW framework functioned as a new tool for occupational therapists to improve occupational therapy practice by supporting their clients’ occupational engagement and promoting better health.

However, there were also barriers to applying the DLW framework in practice. First, because of their practice environment, occupational therapists experienced some challenges in applying the different DLW concepts in their practice. Lack of time, treatment space, and human resources prevented them from incorporating the framework in their occupational therapy practice, such as exploring different dimensions of experience within a restricted time and having limited physical space that hindered the way they wanted to use the DLW framework. Similarly, cross-sectional research regarding facilitators and barriers of occupation-based practice also identified a lack of space and time as a barrier to occupation-based practice (Lloyd et al., 2019) although using occupation to promote clients’ overall health is a core tenet of occupational therapy practice, and occupational therapists implement occupation-based practice (Wilcock & Hocking, 2015). Thus, occupational therapists who want to incorporate the DLW framework may confront environmental restrictions based on their practice settings.

Furthermore, the DLW framework does not provide a step-by-step guideline for specific populations; as a result, some participants experienced difficulty conceptualizing and applying the DLW concepts in practice. Occupational therapists may need to customize the application of the DLW framework according to their clients’ needs; they may feel uncomfortable using the concepts about which they feel uncertain. Thus, we believe there should be ongoing learning opportunities for occupational therapists who seek support in using the DLW framework, in addition to an increased focus on the application during any workshops designed to support occupational therapists’ understanding of the framework. There are different ways to provide ongoing training, such as supervision and refresher training (O’Donovan et al., 2018). The DLW team would offer regular meetings with workshop participants so that people who have been using the DLW framework can share their experiences and ideas with those who have not had a chance to use the DLW framework yet. We understood that participants wanted to learn about application examples in their practice settings. Thus, learning specific examples of how an occupational therapist uses the DLW framework in a particular practice setting would allow therapists to better understand how to apply its concepts in their own practice. In addition, occupational therapists do not need to use all concepts of the DLW framework, but this message might not have been well translated to the participants during the workshops. Thus, future educational opportunities would make sure to emphasize the flexibility in using the DLW framework in practice. There is also room for modification of the DLW framework itself. Some participants in this study said it was not easy for them to apply the DLW concepts with young children and people with limited functional abilities. For example, activities to manage housing and financial stability may be irrelevant for children and people who do not have the ability to manage their financial status. The DLW framework could be amended to add explanations on how to link each concept to these populations by updating available evidence on each section of the DLW framework.

The literature specified there is no single factor that influences learners to incorporate their knowledge into practice (Grol et al., 2007), and we have demonstrated the importance of a multilevel framework that considers different factors influencing knowledge implementation. For example, one participant was aware of the value of the DLW framework but reported that she lacked confidence in her knowledge of the framework. Another participant used the DLW framework because she believed it would improve her clinical practice despite finding the occupational therapy jargon used in the framework challenging. This study demonstrated that different levels of factors influence the adoption of new knowledge. Therefore, future educators will need to identify facilitators and challenges at various levels to promote learners’ application of new knowledge in practice.

### Limitation

Although researchers in this study were able to consider the client-level factors influencing the DLW adoption by understanding the occupational therapists’ experiences and perspectives, we did not directly explore clients’ experience of the DLW framework. Learning about clients’ experiences would take time, but understanding them would be key to evaluating the success of DLW framework in the application (Kirkpatrick & Kirkpatrick, 2006). Therefore, future researchers could evaluate clients’ outcomes and examine their experiences in using the DLW framework.

Some participants were not able to use the DLW framework in practice because of practice changes and stress caused by the COVID-19 pandemic beyond our control. Had COVID-19 not occurred, more occupational therapists may have used the DLW framework in practice. Therefore, after the COVID-19 situation has stabilized, follow-up studies could explore participants’ experiences with the application of the DLW framework.

## Conclusion

Overall, the findings support the benefits of incorporating the DLW concepts in occupational therapy practice by emphasizing the importance of participating in various occupations for people's health and wellness. The DLW framework also benefits occupational therapists by allowing them to better explain what occupational therapists do. The DLW team should support occupational therapists’ application of the DLW framework by providing continuous support after educational workshops, such as regular meetings with workshop participants to share their experiences of using the DLW framework.

## Key Messages

- The DLW framework may improve occupational therapy practice by focusing on the importance of occupation for an individual's health and wellness.
- The DLW team would need to provide ongoing training opportunities to support occupational therapists applying the DLW framework in practice.
